# Hydroxysafflor yellow A improved retinopathy via Nrf2/HO-1 pathway in rats

**DOI:** 10.1515/biol-2022-0030

**Published:** 2022-03-24

**Authors:** Zhihui Sun, Yuanyuan Wang, Rui Xu, Shitong Zhang, Hongtao Yang, Jingjing Song, Tao Chang

**Affiliations:** Department of Ophthalmology, Chengde Central Hospital, Guangren Street, Chengde City, Hebei Province 067000, China; Department of Digestology, Chengde Central Hospital, Guangren Street, Chengde City, Hebei Province 067000, China; Department of Hematology, Chengde Central Hospital, Guangren Street, Chengde City, Hebei Province 067000, China; Department of Osteology, Chengde Central Hospital, Guangren Street, Chengde City, Hebei Province 067000, China

**Keywords:** HYSA, Nrf2/HO-1 signaling pathway, diabetes mellitus, retinopathy

## Abstract

The aim of the study was to investigate the inhibitory effect of hydroxysaff yellow A (HSYA) on diabetic retinopathy (DR). For this, a total of 27 rats were randomly divided into normal control, model, and HSYA groups. The body weight, blood glucose, and blood–retinal barrier damage of the rats were observed and compared. The pathological change of retinal tissue were measured using H&E staining. The apoptosis of retinal tissue ganglion cells was detected by TUNEL. The interleukin (IL)-1β and tumor necrosis fator (TNF)-α levels were detected using enzyme-linked immunosorbent assay. The level of malondialdehyde (MDA) was detected using thiobarbituric acid method. Superoxide dismutase levels were detected using xanthine oxidase method; Nrf2 and total HO-1 protein expressions were detected using western blot assay; Bcl-2 and P53 protein expression was measured using immunohistochemical staining. The body weight and retinal damage of the HYSA group were significantly improved (*p* < 0.01, respectively). The apoptosis index of the HYSA group was lower than the model group (*p* < 0.001). The IL-1β, TNF-α, and MDA levels of the HYSA group were significantly improved in comparison with those of the model group (*p* < 0.01, respectively). The Nrf-2, HO-1, Bcl-2, and P53 protein expression of HYSA group was significantly improved (*p* < 0.001, respectively). In conclusion, HYSA can effectively alleviate the apoptosis of retinal ganglion cells in type 2 diabetic rats and improve the progression of DR.

## 1 Introduction

Diabetic retinopathy (DR) is one of the most common microvascular diseases and also an important ocular complication in diabetic patients. The main characteristics of DR include abnormalities in various cellular functions and structures, such as the ganglion cell apoptosis, angiogenic neuroinflammatory injury, and blood–retinal barrier breakdown [1,2]. Patients with DR can experience rapid deterioration of vision, even blindness, which seriously affects the patients’ quality of life and imposes a great burden on the families and society. Currently available treatments for DR include retinal photocoagulation and vitreous surgery, which are only applicable for patients with advanced diseases. Although these approaches block the progression of the disease, a visual impairment that has occurred in patients cannot be reversed. Therefore, an early intervention and treatment in DR patients is of great clinical significance.

Safflower is a dry tubular flower of *Carthamus tinctorius* L., a plant of the Compositae family. It is a famous herbal medicine commonly used for improving blood circulation, removing blood stasis and treating cardiovascular and thrombotic diseases [3,4]. Hydroxy safflor yellow A (HSYA) is the most important bioactive ingredient of safflower. Pharmacological studies have shown that HSYA has significant cardiovascular and cerebrovascular protective effects owing to its vasodilatation [5], antioxidant [6], and anti-inflammatory activities [7]. HSYA inhibits the lipopolysaccharide- or hypoxia-induced expression of inflammatory factors such as tumor necrosis fator-α (TNF-α) as well as the activity of NF-κB to exert the anti-inflammatory effects [8,9,10]. However, whether HSYA affects DR in the same way remains unclear. On this basis, this study was designed to explore the effect and the underlying mechanism of HSYA on DR in type 2 diabetic rats.

## 2 Materials and methods

### 2.1 Materials

#### 2.1.1 Animal models and grouping

Twenty-seven healthy SPF Wistar rats, weighing 300 ± 20 g, were purchased from Junke Biological Co., Ltd (Nanjing, China) and housed at room temperature (RT) (22 ± 2°C) and relative humidity of 60 ± 5% with a light–dark cycle of 12:12 h and free access to drinking water and diet. These rats were randomly divided into the normal control (NC) group (*n* = 9), the model group (*n* = 9), and the HYSA group (*n* = 9). All rats were adaptively fed for 7 days before inducting in the experiments.


**Ethical approval:** The research related to animal use has been complied with all the relevant national regulations and institutional policies for the care and use of animals.

#### 2.1.2 Reagents

Streptozotocin (STZ) (Sigma, USA); HYSA, purity >98% (National Institutes for Food and Drug Control, China); TUNEL Apoptosis Kit (Boster Biological Technology, Wuhan, China); anti-OH-1, B lymphoblastoma-2 (Bcl-2), nuclear factor-erythroid 2-related factor-2 (Nrf2), and P53 antibodies (Abcam, UK); TNF-α and interleukin-1β (IL-1β)) enzyme-linked immunosorbent assay (ELISA) kits (Abcam, USA).

## 3 Methods

### 3.1 Modeling and drug administration

All procedures were conducted according to the methods previously described [11,12] along with results from preliminary experiments. To establish type 2 diabetes mellitus (T2DM) models, rats in the model group and the HYSA group were briefly injected with STZ in the left lower abdominal cavity (100 g/L STZ in citrate solution, pH 4.5. Injection dose was 60 mg/kg body weight), and those in the NC group were injected with the same volume of citrate solution. Blood glucose was measured from venous blood from the tails 72 h after STZ injection using a blood glucose meter. If urine glucose level was ≥ ++++, blood glucose >16.5 mmol/L, and volumes of urine and drinking water increased significantly, and T2DM model was successfully established. One week after model establishment, animals in the HYSA group were intraperitoneally injected with 50 mg/kg HSYA daily for 6 weeks (daily dose of HYSA injection in human was converted into a bioequivalent dose for rats and the experimental dosage was determined in preliminary experiments). Rats in the NC group and the model group were intraperitoneally given an equal volume of normal saline per day.

### 3.2 Blood glucose measurement

At the end of the experiment, the body weight of the rats was recorded. After fasting for 12 h, blood was taken from their tails and blood glucose was determined using an automatic blood glucose analyzer for animals in each group.

### 3.3 Specimen preparation

At the end of the experiment, after fasting for 12 h, the rats were killed by intraperitoneal injection of 1% pentobarbital sodium (50 mg/kg) and their eyeballs were dissected rapidly. One eyeball was immersed in 4% paraformaldehyde fixed solution for 24 h, and apoptosis was programmed using TUNEL assay, H&E and immunohistochemical (IHC) staining; the other eyeball was rapidly placed at −80°C to measure the levels of IL-1, TNF, and MDA, and superoxide dismutase (SOD) was used for western Blot (WB) assay.

### 3.4 H&E staining

Retinal tissue was removed and fixed in 4% paraformaldehyde solution. The fixed tissue was embedded in paraffin and then sectioned. After H&E staining, the sections were observed under a microscope and the pathological damage of the retinal tissue was analyzed for animals in each group.

Huang et al. [13] pathological scoring criteria were used to evaluate the pathological score in our study. Six sections were taken from each sample, and five high power visual fields (×400) were taken from each section under a light microscope to score and analyze the pathological changes of retina. The scoring criteria of retinal medical records were normal (0 point); rod cell, and cone cell destruction (1–2 points); outer nuclear layer failure (3–4 points); Kernel layer corruption (5–6 points); and ganglion cell layer destruction (7 points).

### 3.5 Detection of ganglion cell apoptosis by TUNEL assay

Retinal tissue was removed and fixed in 40 g/L paraformaldehyde solution at 4°C for 24 h. After removal of the anterior segment and vitreous body, the remaining retinal tissue was embedded in paraffin, sectioned, dewaxed with xylene, and rehydrated with ethanol gradients. After washing three times with public broadcasting service (PBS), the tissue sections were placed in protein kinase K solution (1:200 ddH_2_O) and incubated for 20 min at RT. The sections were washed twice with ddH_2_O, and TdT plus fluorescein-labeled dUTP reaction solution was added, followed by incubation at 4°C overnight. Around 50 μL blocking solution was added and incubated in a humid box at 37°C for 30 min. After washing with PBS for three times, DAPI was added, and sections were mounted with 20% glycerol buffer (v/v). Each specimen was observed using light microscopy and examined under 9 microscopic fields with a digital camera (AxiocamMRc; Zeiss AG) attached to a microscope (magnification, ×200; Axioskop 2 Plus Zeiss AG) to evaluate apoptosis cell rats (nucleus of apoptotic cells was brown).

### 3.6 Determination of IL-1β, TNF-α, MDA, and SOD levels in the retinal tissues of animals in each group

A part of the retinal tissue was taken and homogenized. The levels of IL-1β and TNF-α were detected using ELISA and MDA with thiobarbituric acid method, and SOD by xanthine oxidase method for animals in each group.

### 3.7 Expression of relevant proteins by WB

A part of the retinal tissue was taken and total protein was extracted using lysis buffer. Protein concentration was determined and protein was then subjected to sodium dodecyl sulfate–polyacrylamide gel electrophoresis. The proteins were then transferred to a membrane. After washing three times with PBS, the membrane was incubated with blocking solution containing nonfat dry milk for 1 h. Then the membrane was incubated with anti-Nrf2 or OH-1 antibodies overnight at RT. The following day, the membrane was washed three times with TBS+Tween and incubated with a corresponding secondary antibody for 1 h at RT, followed by washing three times with TBST. Finally, electrochemiluminescence method was used to determine luminescent intensities. The target band was examined using ImageJ software (National Institutes of Health) for gray value analysis.

### 3.8 Detection of Bcl-2 and P53 protein expression in the retina by IHC staining

Paraffin sections were prepared followed by dewaxing and rehydration to water, microwave antigen retrieval in citric acid pH6.0, hydrogen peroxide blocking, and washing with PBS pH 7.2. Then, primary antibody (diluted at 1:100) was added and incubated, and after washing with PBS, the secondary antibody was added and incubated. Diaminobenzidine color development and subsequent hematoxylin counterstaining were conducted. Sections were then observed under a light microscope and images were taken. IHC positive images were semiquantitatively analyzed using the Imagepro-Plus 7.0 image analysis system (MEDIA CYBERNETICS, USA): retina on the section was chosen using 400× magnification, and the integrated optical density (IOD) of this area was measured. Area of the effective region was selected and measured for statistical analysis, and mean IOD/area or mean density was calculated. Three sections per animal and five fields per section were selected for each animal and their mean value was used for analysis.

### 3.9 Statistical analysis

All variables analyzed in this study were continuous ones and measurement data were presented as mean ± SD. One-way analysis of variance was used to compare the overall differences among the NC group, the model group, and the HSYA group, and the test for pairwise comparisons. All statistical analyses in this study were performed using SPSS 23.0 software (SPSS Inc., Chicago, Ill., USA) with a significance level of 0.05.

## 4 Results

### 4.1 Changes in body weight and blood glucose in animals of each group

**Figure 1:**
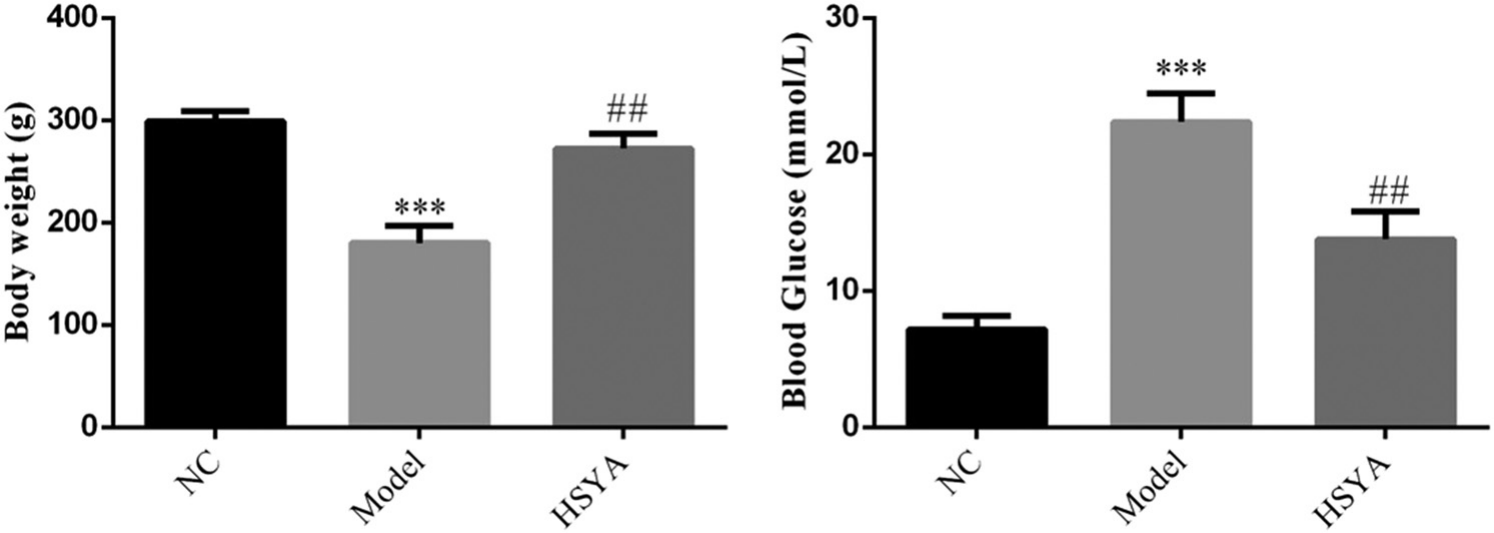
The body weight and blood glucose of different groups. NC: normal control group; model: DR model group; HYSA: the rats were treated with 30 mg/kg HYSA every day. ***: *p* < 0.001, compared with the NC group; ##: *p* < 0.01, compared with the model group.

Body weight of the rats was decreased and blood glucose was increased in the model group compared with that of the NC group (all *p* < 0.001), and the body weight was increased and blood glucose was decreased in animals of the HYSA group compared with that of the model group (*p* < 0.01, respectively, Figure 1).

### 4.2 Pathological observation of H&E staining in rat retina

**Figure 2:**
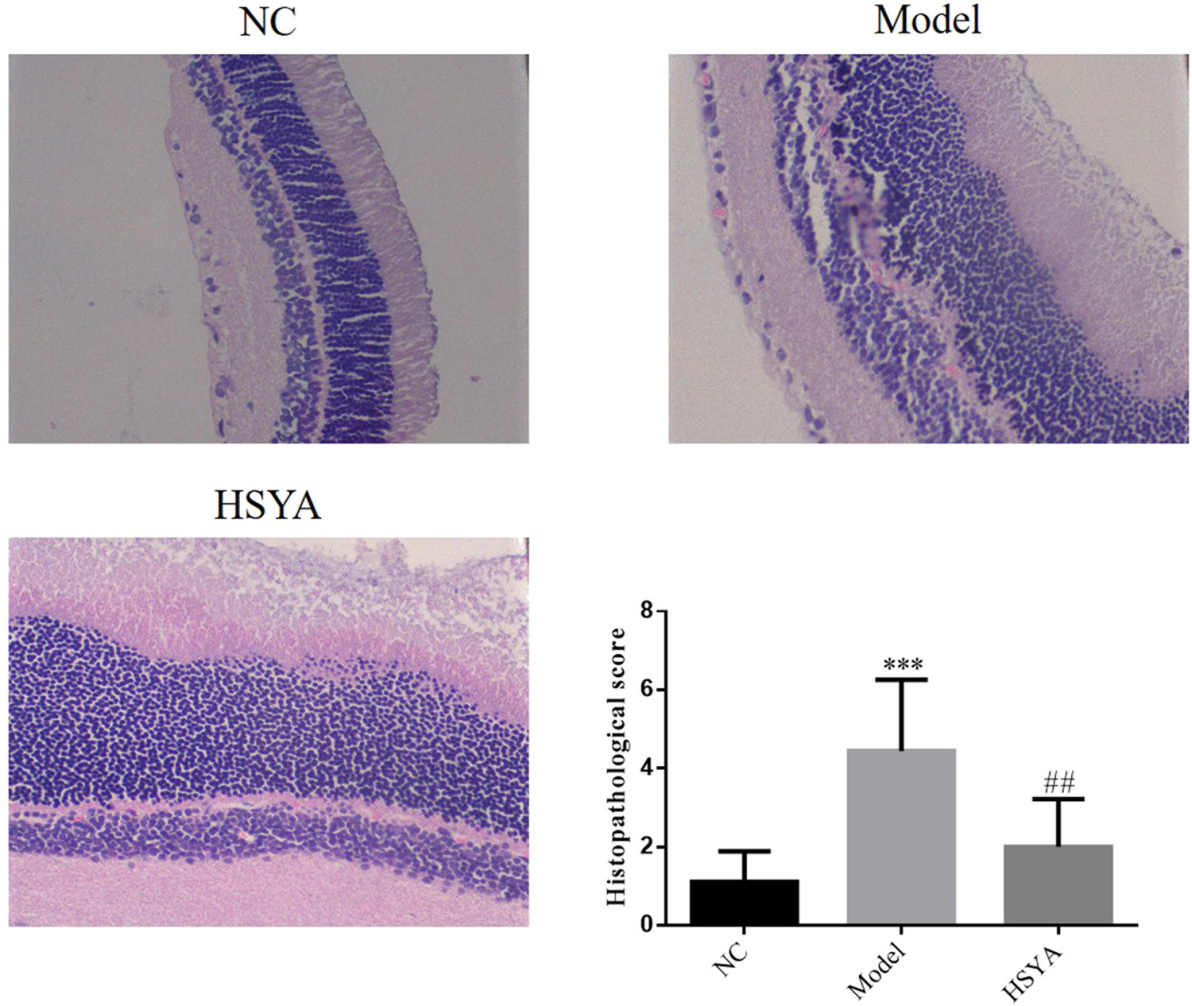
The pathological change of different groups in retina by H&E staining (400×). NC: normal control group; model: DR model group; HYSA: the rats were treated with 30 mg/kg HYSA every day. ***: *p* < 0.001, compared with NC group; ##: *p* < 0.01, compared to the model group.

In the NC group, 10 intraretinal layers were arranged neatly and the morphology of the retinal cells was normal; in the model group, retinal cells were deranged and the thickness of retina was reduced, with endocapillary proliferation, thickened basement membrane, reduced ganglion cells, and vacuolar degeneration in the nerve fiber layer. The histopathological score was significantly upregulated (*p* < 0.001, Figure 2), and in the HYSA group, retinal thickness was slightly thinned with no significantly increased capillary basement membrane. When compared with the model group, the histopathological score had significantly improved (*p* < 0.01, Figure 2). The number of ganglion cells was slightly reduced and slight vacuolar degeneration was seen in the nerve fiber layer. Retinal cells in the inner and outer nuclear layers were arranged neatly, as shown in Figure 2.

### 4.3 Apoptosis of ganglion cells by TUNEL assay

**Figure 3:**
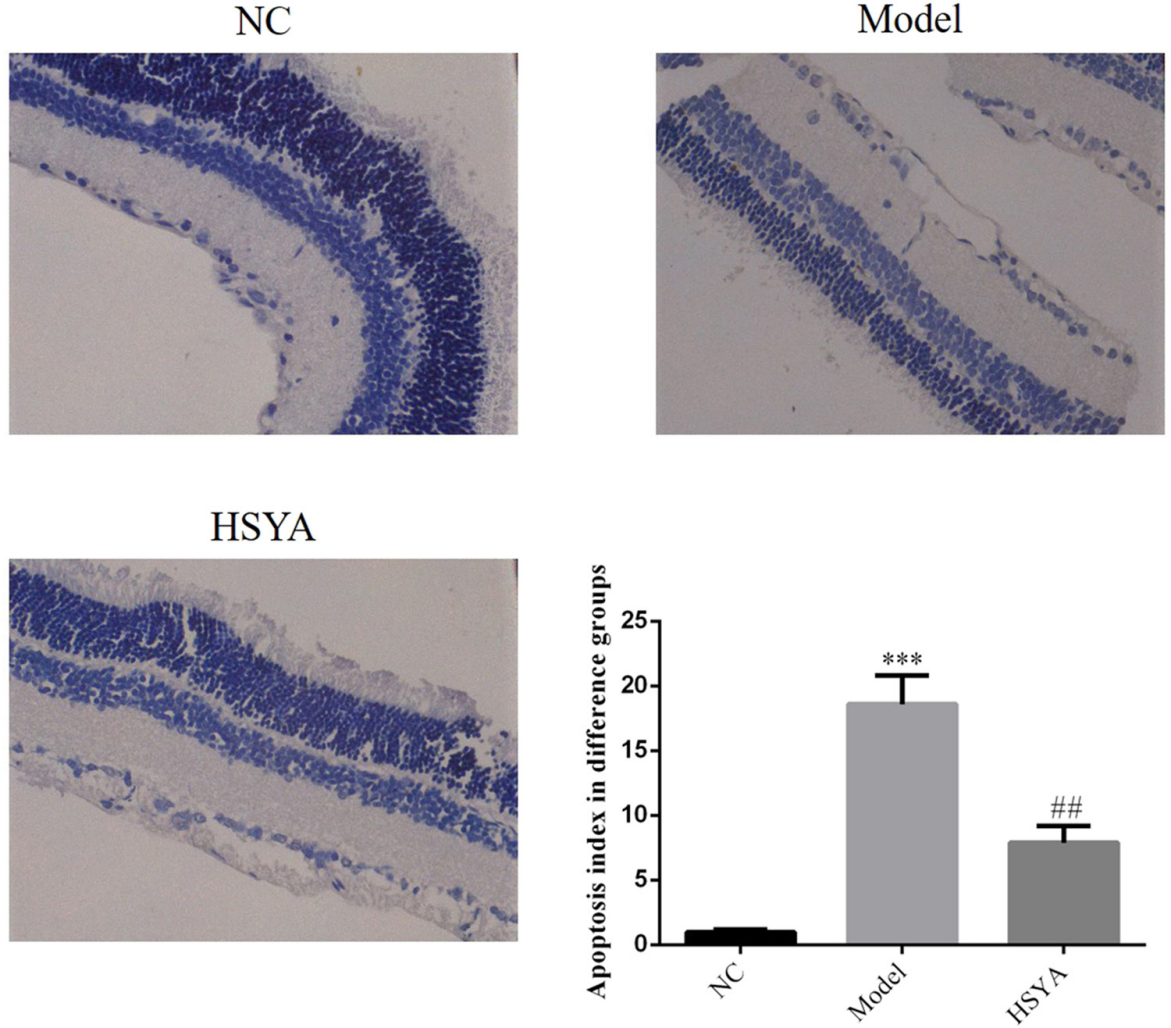
The apoptosis index of different groups in retina by TUNEL assay (400 ×). NC: normal control group; model: DR model group; HYSA: the rats were treated with 30 mg/kg HYSA every day. ***: *p* < 0.001, compared to the NC group; ##: *p* < 0.01, compared with model group.

The apoptosis index of ganglion cells in the model group was significantly increased in comparison with that of the NC group (*p* < 0.001); and after HYSA treatment, the apoptosis index of ganglion cells in the HYSA group was significantly reduced compared with that of the model group (*p* < 0.01, Figure 3), indicating that HYSA could effectively improve the damaged retinal ganglion cells induced by DR.

### 4.4 Comparison of IL-1β, TNF-α, MDA, and SOD levels in retinal tissue of rats between groups

**Figure 4:**
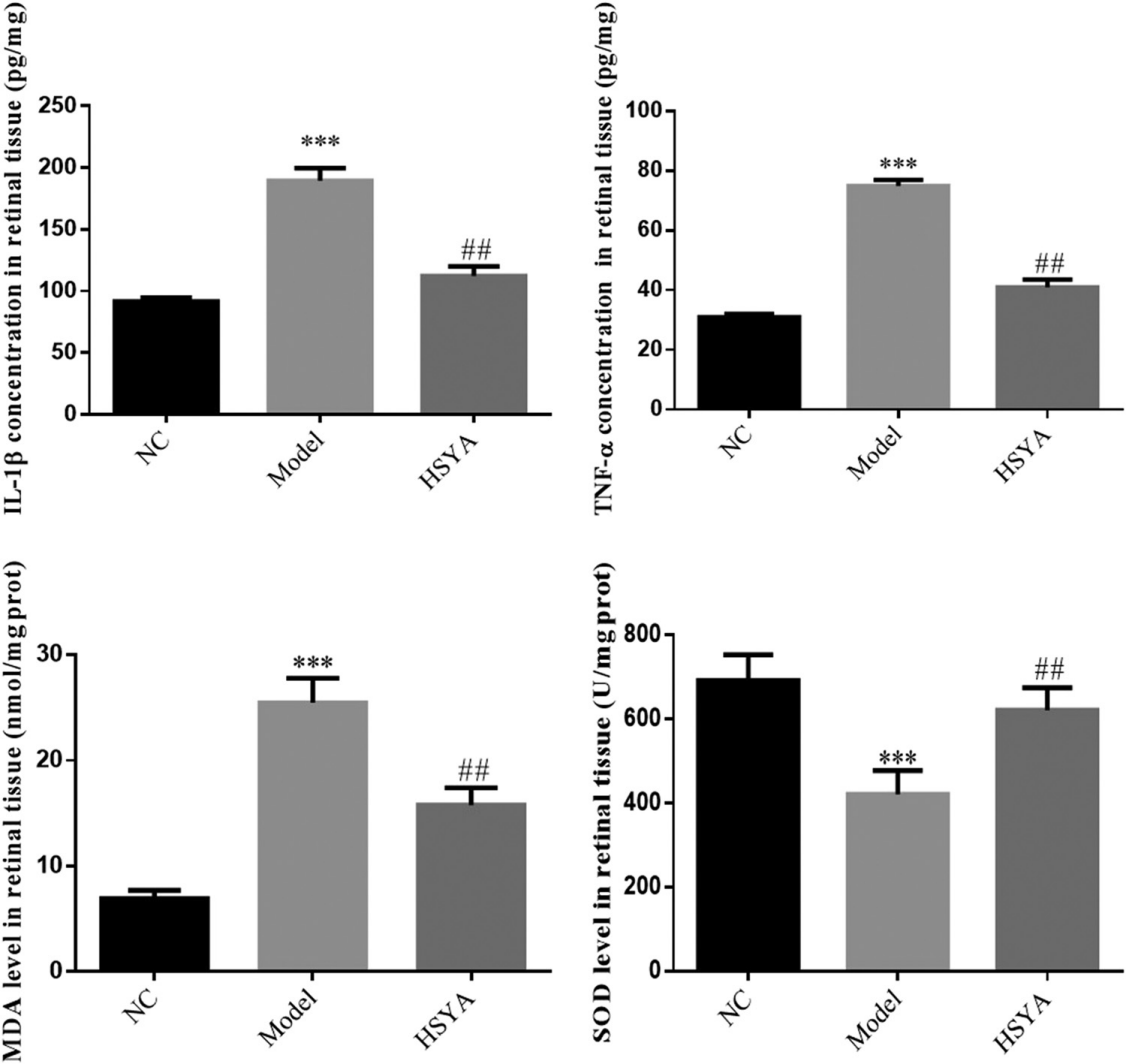
The IL-1β, TNF-α, MDA, and SOD concentrations of different groups. NC: normal control group; model: DR model group; HYSA: the rats were treated with 30 mg/kg HYSA every day. ***: *p* < 0.001, compared to the NC group; ##: *p* < 0.01, compared with model group.

The retinal levels of IL-1β, TNF-α, and MDA were increased and that of SOD was reduced in the model group compared with those of the NC group, with statistically significant differences (*p* < 0.001, respectively). The retinal levels of IL-1β, TNF-α, and MDA were decreased and that of SOD was increased in the HYSA group compared with those of the model group and the differences were statistically significant (*p* < 0.01, respectively, Figure 4).

### 4.5 Expression of Bcl-2 and P53 proteins in rat retinal tissue

**Figure 5:**
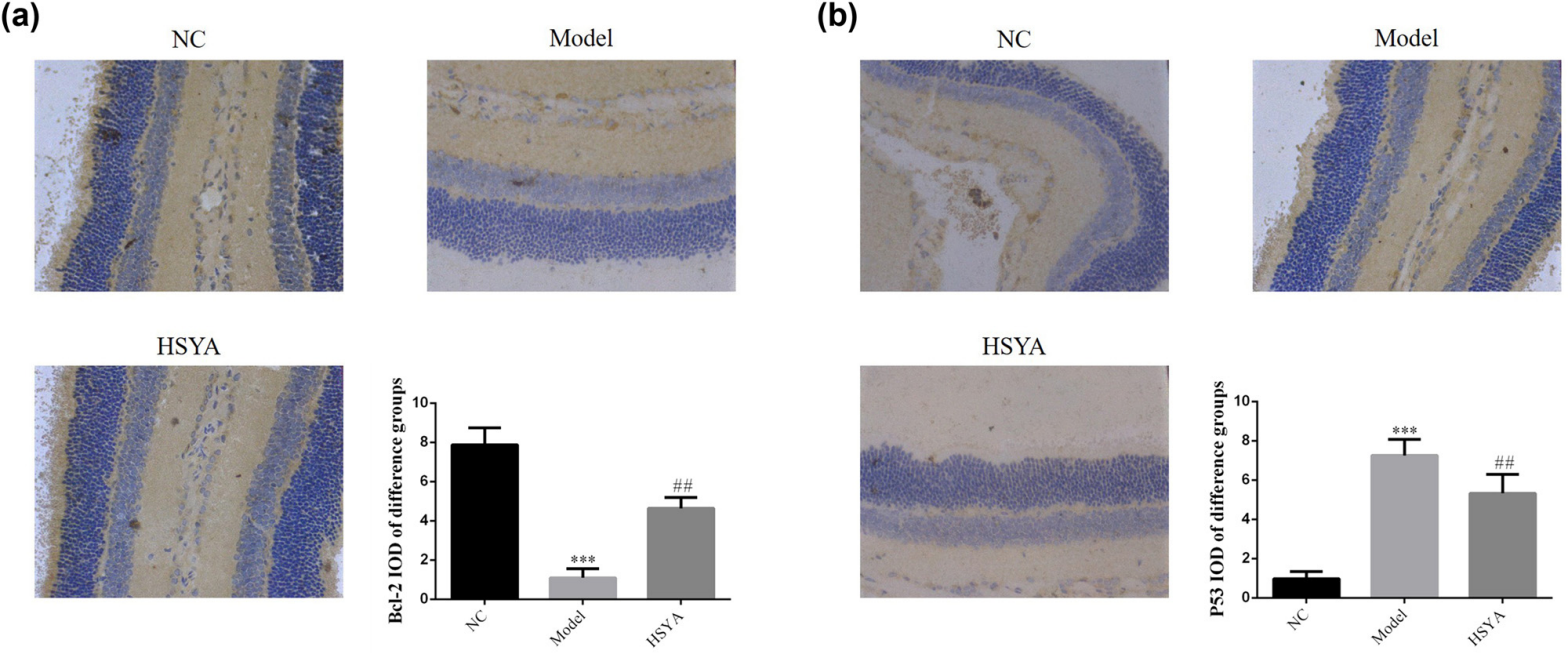
The Bcl-2 and P53 protein expressions of different groups by IHC assay (400×). (a) Bcl-2 protein expression of different groups by IHC assay. NC: normal control group; model: DR model group; HYSA: the rats were treated with 30 mg/kg HYSA every day. ***: *p* < 0.001, compared with the NC group; ##: *p* < 0.01, compared with the model group. (b) P53 protein expression of different groups by IHC assay. NC: normal control group; model: DR model group; HYSA: the rats were treated with 30 mg/kg HYSA every day. ***: *p* < 0.001, compared with the NC group; ##: *p* < 0.01, compared with the model group.

IHC analysis showed that the expression of retinal Bcl-2 protein was significantly decreased and that of P53 was significantly increased in the model group in comparison to those of the NC group (*p* < 0.001, respectively), and after HYSA treatment, the expression of retinal Bcl-2 protein was significantly increased and that of P53 was significantly decreased in the HYSA group in comparison to those of the model group (*p* < 0.001, respectively, Figure 5).

### 4.6 Expression of retinal Nrf-2 and HO-1 proteins in rats of each group by WB assay

**Figure 6:**
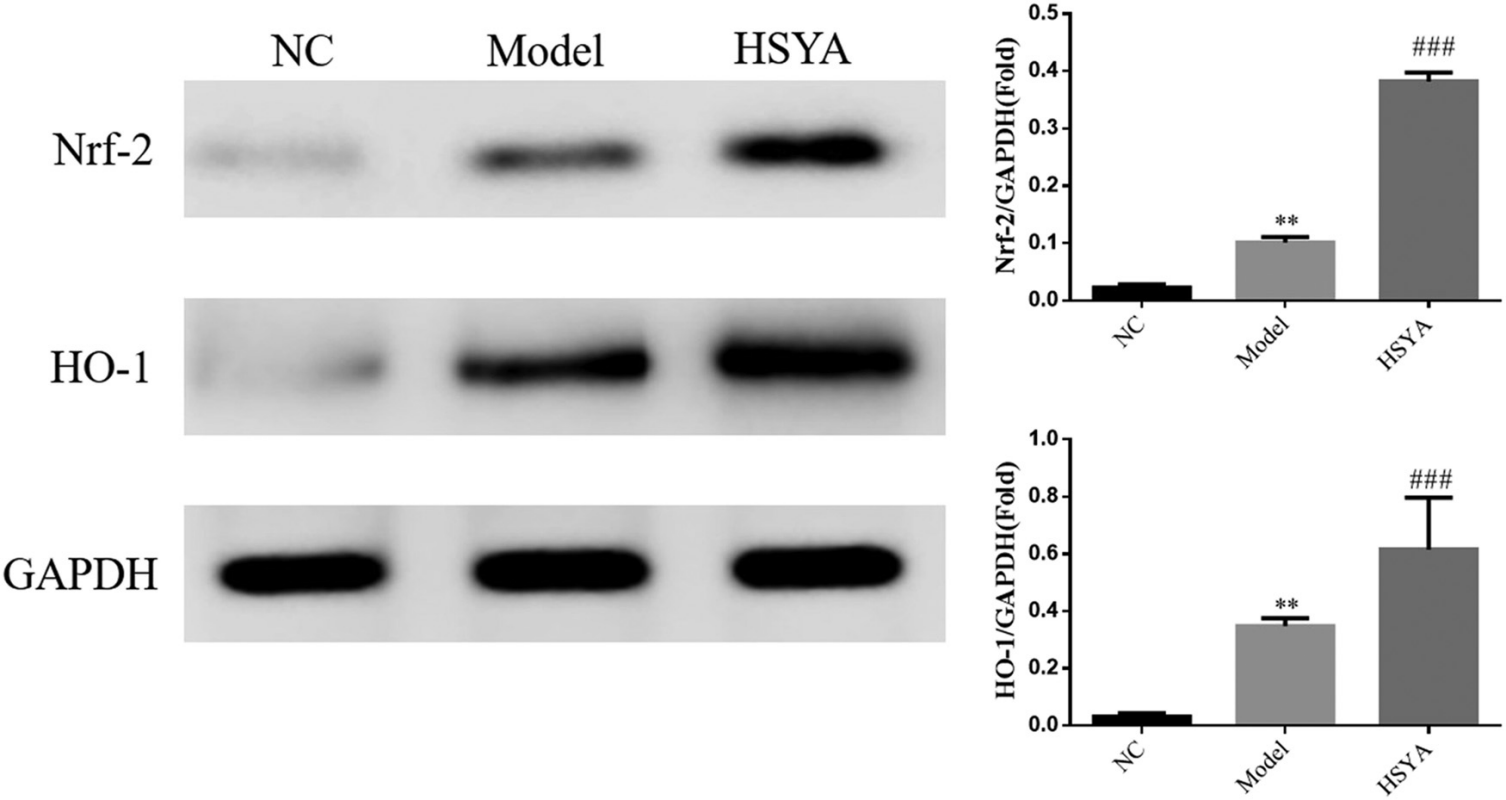
The Nrf-2 and HO-1 proteins expressions of different groups by WB assay. NC: normal control group; model: DR model group; HYSA: the rats were treated with 30 mg/kg HYSA every day. ***: *p* < 0.001, compared to the NC group; ##: *p* < 0.01, compared with the model group.

WB assay showed that compared with the NC group, the expression of retinal Nrf-2 and HO-1 proteins in animals of the model group was significantly increased (*p* < 0.001, respectively). After HYSA intervention, the expression of retinal Nrf-2 and HO-1 proteins in the HYSA group was significantly increased (*p* < 0.001, respectively, Figure 6).

## 5 Discussion

DR is one of the common microvascular complications of diabetes, which may lead to visual loss. DR seriously affects patients’ quality of life and imposes a heavy burden on families. The pathogenesis of DR has not been completely established to date. Studies have reported that the pathogenesis of DR is closely related to diseases including vasculopathy, neuropathy, and inflammation, among which, the inflammatory response and oxidative stress mediated by Nrf-2/HO-1 signaling pathway may play an important role in this process [13]. Nrf-2 is an important transcriptional regulator that promotes the transcription and expression of various antioxidant stress-related protein genes such as catalase, heme oxygenase-1, and glutathione S-transferase to further eliminate a large number of oxidative stress products and inflammatory factors in the body, reducing the inflammatory damage of tissues and organs.

Inflammatory cytokines are the initiating factors for the pathways above. Studies [11,14] have shown that IL-1β, TNF-α, and other inflammatory cytokines are highly expressed in the retina of DR patients, which contributes to the inflammatory response and damage to the blood–retinal barrier and promotes the development of DR. TNF-α, among others, is mainly activated by macrophages or monocytes, and its expression is positively correlated with the destruction of blood–retinal barrier and vascular cell death. Therefore, TNF-α is believed to be the initiator for DR inflammatory response and can be used as an indicator for DR severity. IL-1β plays an important role in the pathogenesis of DR. IL-1β is highly expressed in DR patients to promote the secretion of other inflammatory factors, thereby enhancing the expression of cell adhesion molecules, calcium overload, and apoptotic pathways and the consequent DR development in diabetic patients. In addition, oxidative stress is also involved in the pathological process of DR [15]. MDA and SOD are important indicators of oxidative stress. MDA is a product of lipid peroxidation and is positively correlated with oxidative stress. Studies have shown that the level of MDA, an oxidative stress index, is significantly increased and that of SOD is significantly decreased in diabetic rats, suggesting that diabetic rats are in an oxidative stress state. Therefore, control of the inflammatory response and oxidative stress damage is one of the important approaches to manage DR.

The results of this study showed that compared with the NC group, the levels of retinal IL-1β, TNF-α, and MDA were increased and that of SOD was reduced in the model group, and the expression levels of Nrf2, HO-1, and P53 proteins were increased and that of Bcl-2 was significantly decreased in the model group, suggesting that significant inflammatory response and oxidative stress damage occurred in the retina of DR rats, which may be related to the activation of Nrf-2/HO-1 signaling pathway.

HSYA is the major bioactive component in safflower, and its antioxidant activities have been proven in recent studies [16,17]. Studies of HSYA in DR, however, are rarely reported. The results of this study showed that compared with the NC group, the body weight of the model group was significantly decreased and blood glucose level, retinal histopathology and ganglion cell apoptosis index were significantly increased in the model group; and the expression level of Bcl-2 was significantly decreased and that of P53 was significantly increased in the model group, suggesting that apoptosis of retinal cells in DR rats was significantly increased with proliferative lesions in retina. These results were consistent with the findings previously reported [18]. However, compared with the model group, the blood glucose, retinal histopathology, and ganglion cell apoptosis index of the HYSA group were significantly reduced, and the expression of Bcl-2 level was increased and that of P53 was decreased in the HYSA group, indicating that HYSA could effectively control the blood glucose level, reduce the damage of retinal barrier, and inhibit the apoptosis of retinal ganglion cells.

The Nrf2/HO-1 signaling pathway plays a crucial role in the antioxidative stress response, and nuclear translocation of Nrf2 is the key to HO-1 activation [19]. WB assay was used in this study to confirm whether HYSA induced an antioxidative stress response via the Nrf2/HO-1 pathway, and the results showed that the expression of Nrf2 and HO-1 proteins in the HYSA group was significantly increased compared with that of the model group (*p* < 0.01), indicating that DR induced retinal tissue damage and HYSA enhanced the expression of HO-1 protein by inducing nuclear translocation of Nrf2 to protect from oxidative damage.

## 6 Conclusion

HYSA can effectively inhibit the inflammatory response and oxidative stress damage of retinal cells in DR (of T2DM) and protect retinal ganglion cells. The underlying mechanism may be related to the activation of the Nrf-2/HO-1 signaling pathway.
